# Learning From Success and Failure: Biologics for Non-approved Skin Diseases

**DOI:** 10.3389/fimmu.2019.01918

**Published:** 2019-08-08

**Authors:** Reinhart Speeckaert, Jo Lambert, Nanja van Geel

**Affiliations:** Department of Dermatology, Ghent University Hospital, Ghent, Belgium

**Keywords:** biologics, off-label, TNF-α, IL-1, IL-12, IL-23, IgE, CD20

## Abstract

The impressive potential of biologics has been demonstrated in psoriasis, hidradenitis suppurativa, and urticaria. Numerous biologicals are entering the field for a restricted number of skin disorders. Off-label use of biologics in other recalcitrant skin diseases has increased. Mounting data point to the potential of already existing biologics acting on the IL-17/IL-23 pathway in skin disorders with epidermal hyperkeratosis (e.g., pityriasis rubra pilaris), acneiform inflammation (e.g., hidradenitis suppurativa), and loss of mucosal integrity (e.g., aphthosis). TNF-α blockers are also effective in the latter conditions but seem of particular value in granulomatous (e.g., granuloma annulare) and neutrophilic disorders (e.g., pyoderma gangrenosum). Failure of IL-17 blockade in skin diseases resulting from immune-mediated cell destruction (e.g., alopecia areata and vitiligo) illustrates its limited involvement in Th1-dependent skin immunology. Overall, disappointing results of TNF-α blockers in alopecia areata and vitiligo point to the same conclusion although promising results in toxic epidermal necrolysis suggest TNF-α exerts at least some *in vivo* Th1-related activities. Acting on both the Th1 and Th17 pathway, ustekinumab has a rather broad potential with interesting results in lupus and alopecia areata. The efficacy of omalizumab in bullous pemphigoid has revealed an IgE-mediated recruitment of eosinophils leading to bullae formation. Reconsidering reimbursement criteria for less common but severe diseases seems appropriate if substantial evidence is available (e.g., pityriasis rubra pilaris). For other disorders, investigator- and industry-initiated randomized clinical trials should be stimulated. They are likely to improve patient outcome and advance our understanding of challenging skin disorders.

## Introduction

A large number of dermatological disorders is mediated by a deregulated skin immune response. In the last 2 decades, biologics have revolutionized the traditional approach by achieving unprecedented results with limited adverse events. Unfortunately, their use is restricted to a very small number of diseases (psoriasis, urticaria, and hidradenitis suppurativa). The promise of a targeted approach triggers clinicians to test these drugs in recalcitrant disorders despite their off-label status. More than 100 articles have been published on the off-label use of biologics. This is in obvious contrast with the limited number of prospective randomized trials that have been carried out. In this review we summarize the evidence of the currently available biologics targeting IL-1, IL-12/IL-23, IL-17, IL-23, TNF-α, CD20, and IgE in different “off-label” skin conditions (Supplementary Table S1).

## Disorders Based on Targeted Immune-Based Cell Destruction: Alopecia Areata and Vitiligo

Given the role of TNF-α in the Th1 response, it made sense to test TNF-α inhibitors in alopecia areata and vitiligo. Nonetheless, adalimumab fails to induce hair regrowth in most alopecia areata patients (1). Several cases have been published mentioning *de novo* development of alopecia areata due to TNF-α blockers (2, 3). However, some successful cases have also been reported (4). The risk of vitiligo is significantly increased in patients receiving this class of biologics [hazard ratio: 1.99 (95% confidence interval: 1.06–3.75)] (5). Overall, repigmentation using TNF-α blockers is limited in vitiligo and this approach was considered to be ineffective (6). Nonetheless, TNF-α inhibitors can be useful to halt disease progression in active vitiligo (7). No data on the combination of TNF-α inhibitors and phototherapy are available.

Increased IL-17 levels and Th17 lymphocytes have been observed in the skin and blood of patients with alopecia areata and vitiligo (8, 9). Unfortunately, most alopecia areata patients did not show any response to secukinumab, an anti-IL17 monoclonal antibody (10). The same result was observed in our trial using secukinumab in progressive vitiligo which showed that 7/8 patients developed new skin depigmentations leading to an early halt of further recruitment. In subsequent experiments, we showed that Th17.1 cells rather than Th17 cells are increased in active vitiligo (11). Th17.1 lymphocytes are a subgroup of Th17 cells gradually differentiating into non-classical Th1 cells. IL-12 and IL-23 (in combination with low TGF-β) are the main cytokines driving Th17 > Th1 polarization. In that regard, ustekinumab, a combined anti-IL12/23 monoclonal antibody, might represent an attractive treatment option. Despite some reports mentioning new onset vitiligo and alopecia areata during ustekinumab, cases showing repigmentation have been published (12). In alopecia areata, some impressive cases of hair regrowth following the initiation of ustekinumab illustrate the interesting potential of this biologic (13, 14). Increased serum levels of IL-23 have been found in vitiligo although no clinical data are available for IL-23 blockers (15).

## Acneiform Disorders With Neutrophils: Hidradenitis Suppurativa and Acne Conglobata

The introduction of TNF-α blockers in the therapeutic arsenal of hidradenitis suppurativa has increased the scientific interest for this disorder. The improvement of abscesses and pustules with beneficial effects on the quality of life are clear although treatment failure is more common compared to psoriasis (16).

IL-17 is considered to be an important factor in the detrimental inflammatory responses in HS. Case reports and a small pilot trial mention clear improvement in HS patients receiving secukinumab (17, 18). In contrast, an *ex vivo* study on lesional skin samples showed a more pronounced decrease in pro-inflammatory cytokine production and antimicrobial peptides with TNF-α inhibitors compared to IL-17 inhibition (19). Treatment with ustekinumab leads to moderate-marked responses in 82% of HS patients (20). In a retrospective analysis, guselkumab showed improvement in 8/11 (73%) of HS patients (21, 22).

In a small randomized controlled trial (RCT) (10:10), anakinra—a recombinant IL-1 receptor antagonist—displayed promising results with a 67% decreased activity score compared to 20% in the placebo arm (23). Nonetheless, some HS patients fail to improve with both anakinra and canakinumab (= monoclonal antibody against IL-1β) (24, 25). Up till now, large RCTs are missing.

Acne conglobata can be a disfiguring disease with limited treatment options in case of failure of systemic retinoids. Sand and Thomsen reported benefit from TNF-α inhibitors in 64% (7/11) of patients with severe refractory acne conglobata (26). Propionibacterium acnes stimulates keratinocytes to produce IL-1α and TNF-α. Additionally, peripheral blood mononuclear cells (PBMCs) of acne patients stimulated with P. acnes produce increased amounts of TNF-α and IL-8 pointing to the central role of TNF-α in this condition (27). P. acnes also promotes Th17 and Th17.1 responses (28) although no data have been published on the efficacy of IL-17 blockers or ustekinumab.

## Systemic Disorders: Lupus and Dermatomyositis

A phase II RCT of patients with active systemic lupus erythematosus (SLE) demonstrated that the addition of ustekinumab to standard care resulted in an improved efficacy (29). After 6 months, a Systemic Lupus Erythematosus Responder Index (SRI)-4 response was achieved in 62% of ustekinumab treated patients compared to 33% in the placebo group. Although some patients have been reported to exhibit lupus-like cutaneous disorders following ustekinumab, several successfully treated cases of subacute and discoid cutaneous lupus can be found in literature with ustekinumab (30–32). Regarding TNF-α blockers, lupus is a well-known adverse event which can display diverse presentation patterns (33).

Rituximab showed promising results in open-label studies but failed to reach the primary endpoints in 2 RCTs (34, 35). However, some confounding factors such as concomitant immunosuppressive medication and problems in study design (e.g., outcome measures with limited sensitivity) might have played a role. Nonetheless, cutaneous lesions of SLE showed beneficial results in a retrospective study. At 6 months, 76% of patients (*n* = 50) showed improvement in mucocutaneous lesions with 40% complete responses. The response to rituximab in subacute cutaneous lupus erythematosus and chronic cutaneous lupus erythematosus seems more variable (36).

Omalizumab was tested in a small RCT (*n* = 15) which enrolled SLE patients with increased levels of IgE anti-dsDNA, anti-Sm or anti-SSA autoantibodies. A significant improvement in Systemic Lupus Erythematosus Disease Activity Index 2000 (SLEDAI-2k) scores was found at 16 weeks although the difference was lower than the clinically minimal important change (37).

A similar pattern emerges with anti-TNF-α antibodies in dermatomyositis which can develop during therapy. Nonetheless, an RCT demonstrated that infliximab can be of value in a subset of patients despite failure to reach the endpoints after 16 weeks (38).

In a retrospective series of patients with refractory idiopathic inflammatory myopathies (including antisynthetase syndrome, dermatomyositis and polymyositis) rituximab resulted in a significantly decreased dependency on glucocorticoids and favorable clinical responses (39).

Abnormal IL-1 receptor antagonist production has been observed in dermatomyositis and polymyositis. 4/15 (36.4%) patients with dermatomyositis or polymyositis improved with anakinra (40).

## Aphthosis (and Behcet's Syndrome)

Anti-TNF-α inhibitors were useful in resistant cases of recurrent aphthous stomatitis. Adalimumab, etanercept and infliximab have all shown good efficacy. Complete responses have been documented in approximately two-thirds of patients treated with adalimumab (41). Adalimumab is also effective to reduce other manifestations of Behcet's syndrome including the resolution of venous thrombosis (42).

Secukinumab showed benefit in five patients with Behcet's syndrome (and concomitant ankylosing spondylitis or psoriatic arthritis) refractory to conventional treatment and anti-TNF-α therapy. Improvement of active mucocutaneous manifestations was evident. Especially the rapid resolution of oral ulcerations was remarkable (43). Different studies have confirmed the involvement of IL-17 in the pathogenesis. Ustekinumab was also effective in a small prospective study of 14 patients. After 12 weeks, complete response was seen in 64%, partial response in 21% with 14% non-responders (44).

IL-1 antagonism (Anakinra) is mainly tested on uveitis in Behcet's syndrome although a small pilot study has investigated the effect on the mucocutaneous complaints. In five of six patients the severity and number of ulcers improved with two cases of complete response (45).

## Neutrophilic Disorders: Pyoderma Gangrenosum (PG)

Infliximab is the only biologic with an RCT for classic PG showing improvement in 20/29 patients (46). Etanercept and adalimumab have variable efficacy in small studies. Paradoxical new onset PG has also been linked to TNF-α inhibitors. Beneficial results have been ascribed to ustekinumab in case reports (47). This is in line with the role of the IL-17/23 pathway in neutrophil migration (48). A retrospective study found complete responses for infliximab in 63.6% (*n* = 33), for adalimumab in 57.1% (*n* = 28), for etanercept in 71.4% (*n* = 7) and for ustekinumab in 66.6% (*n* = 9) of patients. These results were all superior to corticosteroids (48.8%; *n* = 78) (49). Anakinra and canakinumab lead to variable outcomes (50, 51).

Occurrence of Sweet syndrome is possible during anti-TNF-α treatment although it can also be effective in pre-existing Sweet syndrome (52, 53). Some isolated cases responded to IL-1 inhibition (anakinra) (54, 55). Neutrophilic dermatoses associated with auto-inflammatory disorders (e.g., Schnitzler syndrome, Muckle-Wells syndrome) respond well to IL-1 inhibitors (56).

## Bullous Disorders: Bullous Pemphigoid (BP) and Other Bullous Diseases

Remarkable improvements have been seen for omalizumab in BP. A systematic review found 22 reported cases with 84% complete responses. Anti-BP180 IgE antibodies correlate with disease activity in BP supporting their pathogenic relevance (57). IgE autoantibodies against BP180 and BP230 were found in, respectively 21/36 (58.3%) and 18/36 (50%) BP sera (58). The favorable safety profile of omalizumab makes it an attractive treatment option for BP (59).

Rituximab (anti-CD20) also led to 85% complete responses in BP (in 62 patients). Rituximab was associated with lower recurrence rates and a longer disease-free period compared to omalizumab (60). Patients that first did not respond to omalizumab but improved using rituximab (61), but also cases with improvement on omalizumab after failure to rituximab have been published.

Development of BP using anti-TNF-α treatment can occur sporadically although this link remains controversial. Both for ustekinumab and secukinumab cases of successful treatment of BP and new onset BP during treatment have been described (62–65). IL-17 production by innate immune cells, especially neutrophils, is characteristic in lesional BP skin. IL-17 upregulates matrix-metalloproteinase-9 and neutrophil elastase expression which are involved in blister formation (66). Elevated serum concentrations of IL-23 were confirmed in BP patients and rising levels were associated with disease relapse. This pathway could therefore be potentially interesting in BP (66).

Besides pemphigus, rituximab has been administered successfully in other autoimmune bullous diseases. In a retrospective cohort, mucous membrane pemphigoid (*n* = 14) exhibited disease control in 85.7% (partial response: 64.3%, complete response: 28.6%) (67). Similarly, some encouraging data have been obtained in ocular cicatricial pemphigoid (68). A meta-analysis showed that rituximab is also a promising option in epidermolysis bullosa acquisita (69).

## Psoriasiform Disorders: Pityriasis Rubra Pilaris

Off-label use of biologics has been extensively performed in PRP given its high clinical and pathogenic similarity with psoriasis. Anti-TNF-α, anti-IL-17 and anti-IL-12/23 treatments are all effective with marked to complete responses ranging around 50–78%. Partial responses were seen in 11–25% of patients and lack of response in 11–25% (70). Infliximab exhibited superior results compared to adalimumab while ustekinumab is currently most widely used for this disorder. Recent case series on IL-17 inhibition (secukinumab and ixekizumab) show a fast clearance and limited risk of recurrence after therapy withdrawal (71, 72). Nonetheless, treatment response is difficult to predict due to the heterogeneity of the disease (73). Both cases with progression using IL-17 inhibition that subsequently improved using ustekinumab (74) as a patient with limited response to ustekinumab who benefited from secukinumab, have been published (73).

## Granulomatous Disorders: Granuloma Annulare

The role of TNF-α producing macrophages in granuloma formation points to a key role of this cytokine in granuloma annulare. A summary of published cases showed that 20/26 patients with granuloma annulare responded to anti-TNF-α treatment (75). Overall, these data support a high efficacy of TNF-α inhibitors in granuloma annulare although well-designed RCTs are missing. Nonetheless, granuloma annulare has also been reported as a side effect of biologics including adalimumab, infliximab and etanercept but also during treatment with secukinumab (76–78).

## Mastocytosis

Given its excellent efficacy in urticaria, the use of omalizumab in other mast cell mediated diseases such as mastocytosis looks promising. Most published cases report excellent efficacy on systemic symptoms (e.g., gastrointestinal problems, pruritus). Some interesting patients with resolution of cutaneous lesions have been observed (79, 80). A multicenter RCT with 7 patients receiving omalizumab and 9 placebo was conducted. After 6 months, improvement in French Association for the Initiatives of Research on Mastocyte and Mastocytosis (AFIRMM) score was higher in the omalizumab group (52–26 vs. 104–102) although significance was not reached (81).

## Toxic Epidermal Necrolysis

Being one of the most life-threatening skin disorders, treatment of toxic epidermal necrolysis is extremely important. TNF-α has been identified in the blister fluid and serum of TEN patients (82). Most evidence is gathered from case series and case reports. Nonetheless, the majority of cases show excellent responses with improved outcomes compared to the expected mortality (83). An interesting case series of 10 patients with excellent outcome using etanercept was published (84). Adequate screening for infections is necessary given the high prevalence of soft tissue infections (*S. aureus, P. aeruginosa, K. pneumoniae*) (83). An RCT comparing etanercept (*n* = 48) with corticosteroids (*n* = 43) showed a lower mortality rate after etanercept (8.3%) compared to predicted outcome (17.7%). The difference with the corticosteroid group (16.3%) was not significant although the time for complete skin healing was lower (85). Nonetheless, more than 50 cases have been described developing TEN despite concurrent treatment with TNF-α blockers.

## Lichen Planus

Lichenoid drug eruptions are a well-known side effect of TNF-α blockade (86). Cutaneous and oral lichen planus, lichen planopilaris and lichen striatus have all developed in patients receiving anti-TNF- α treatment. Nonetheless, in some cases of extensive lichen planus adalimumab showed improvement and isolated cases of oral lichen planus and nail lichen planus improved using etanercept (87–89). Despite some promising reports, rituximab failed to show efficacy in a small trial with five consecutive patients with erosive lichen planus (90). Data on other biologics are very limited.

## Discussion

**Figure 1:**
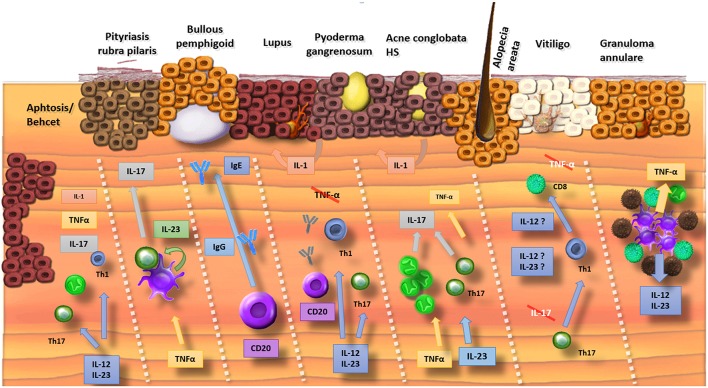
Summary of the involved cytokines in cutaneous disorders (Crossed interleukins: trials were conducted but failed). Mucosal ulceration involves a wide range of cytokines and therefore biologics inhibiting different pathways (IL-1, TNF-α, and IL-17/23) result in improvement. Pityriasis rubra pilaris has a psoriasis-like pathophysiology with a TNF-α stimulated production of IL-23 by dendritic cells. This results in the release of IL-17. Bullous pemphigoid is an antibody-mediated disorder where both inhibition of B lymphocytes (e.g., rituximab) as binding of IgE-antibodies with omalizumab is efficacious. The pathophysiology of lupus is complex with a possible driving role for IL-12 and IL-23. Mixed results for inhibition of B-lymphocytes with rituximab were obtained. TNF-α inhibitors are associated with drug-induced lupus. In neutrophilic pustular disorders (pyoderma gangrenosum, acne, hidradenitis), the IL-17 pathway seems crucial with beneficial results if cytokines of this pathway are targeted (IL-17, IL-23, and TNF-α). In alopecia areata and vitiligo, antibodies against TNF-α and IL-17 were disappointing. Some evidence exists for a role of ustekinumab (IL-12/23) in alopecia areata. TNF-α and IL-12/23 can be targeted in granuloma annulare.

Several promising results have been obtained by the off-label use of biologics and by pilot trials (Table 1, Figure 1) although disappointing results can be equally important to clarify the underlying pathogenic pathways. Failure of both TNF-α inhibitors and IL-17A-blockers in alopecia areata and vitiligo limits the involvement of these cytokines in the immune-mediated destruction of skin cells (10, 11). However, the apparent efficacy of TNF-α inhibitors in toxic epidermal necrolysis and occasional cases of alopecia areata and vitiligo suggest that the role of TNF-α is more complex (4, 7, 91). Alopecia areata and vitiligo remain biologic orphan diseases although some exciting cases have been published showing hair regrowth after initiation of ustekinumab (13, 14).

**Table 1:** Biologics and their efficacy in off-label skin diseases.

	**Anti-TNF-α**		**Anti-IL12/23**		**Anti-IL17**		**Anti-IL23**		**Anti-IL1**		**Anti-IgE**		**Anti-CD20**	
	**Efficacy**	**Evidence**	**Efficacy**	**Evidence**	**Efficacy**	**Evidence**	**Efficacy**	**Evidence**	**Efficacy**	**Evidence**	**Efficacy**	**Evidence**	**Efficacy**	**Evidence**
Alopecia areata	–	CR	+	CR	–	PT								
	AE	CR												
Vitiligo	–/(+)	PT			–	PT								
	AE	RCS												
Hidradenitis suppurativa	+	RCT	+	PT	+	CR			+	RCT				
Acne conglobata	+	CR												
Lupus	AE	CR	+	RCT					+/–	PT, CR	+/–	RCT	+	CR, PT
													–	RCT
Dermatomyositis	+/–	RCT							+/–	CR			+	CR
Aphthosis/behcet	+	RCT	+	PT	+	CR			+	CR				
Pyoderma gangrenosum	+	RCT	+	CR					+	CR				
	AE	CR												
Bullous pemphigoid	AE	CR	AE	CR	AE	CR					+	CR	+	CR
			+	CR	+	CR								
Pityriasis rubra pilaris	+	PT	+	PT	+	CR								
Granuloma annulare	+	PT, CR												
Toxic epidermal necrolysis	+	RCT											AE	CR
Lichen planus	+	CR											–	PT
	AE	CR											+	CR

*Evidence: CR (white), case reports (at least 2 case reports); PT (gray), pilot trials; RCT (black), randomized controlled trials; RCS, retrospective cohort study; + (green), positive evidence; +/**–** (orange), unclear/conflicting results; – (red), no clear benefit; AE (blue), reported as adverse event*.

Promising early data concerning the blockage of the IL-23/IL17 pathway in HS confirm that this pathway is an important initiator of a suppurative neutrophilic inflammation (19, 20, 22, 92). The benefit of blocking IL-17 in aphthosis is in agreement with its defensive capacities against mucosal invasion of pathogens and its protective actions to maintain epithelial barrier integrity.

The results of the phase II trial of ustekinumab in lupus could be a major breakthrough although phase III trials have to be awaited. Increased IL-12 and IL-23 levels have been reported in SLE patients and genetic research links the IL-12 pathway with an increased susceptibility for SLE (29). In general, interferon is considered the main driving factor in lupus. In contrast, the efficacy of ustekinumab was independent of interferon levels (29, 31, 32).

One of the most remarkable findings was undoubtedly the impressive resolution of bullae in BP following the administration of anti-IgE antibodies (59, 59, 60, 93, 94). This confirms the pathogenic role of anti-IgE antibodies which seem actively involved in the development of bullae. Mice injected with IgE autoantibodies from BP patients in grafted human skin developed erythema and infiltration of eosinophils in the skin and ultimately histological dermal-epidermal separation (95). Further studies on omalizumab in bullous pemphigoid seem of particular value given its efficacy and improved safety profile compared to the alternative options.

While current biologics cover mainly the Th17 pathway, strong inhibition of important Th1 cytokines such as IFN-γ is not (yet) available leading to disappointing results in vitiligo and alopecia areata. Dupilumab, a monoclonal antibody directed against IL-4 and IL-13, developed for atopic dermatitis interferes with key cytokines in the Th2 pathway and offers new possibilities for off-label use. Case reports of good response to different types of chronic itch including prurigo nodularis, uremic pruritus and genital pruritus illustrate the interesting potential of dupilumab (96–99). New development of alopecia areata has been observed during treatment with dupilumab (100–102). Nonetheless, patients with both atopic dermatitis and alopecia areata treated with dupilumab have repeatedly demonstrated hair regrowth (103–105). Furthermore, some isolated cases were published with beneficial results in bullous pemphigoid (106), eosinophilic annular erythema (107), and papuloerythroderma of Ofuji (108).

Biologics have been linked to the occasional development of alopecia areata, vitiligo, lupus, dermatomyositis and bullous diseases. However, as we have learned from TNF-α antagonists in psoriasis, this finding does not exclude their efficacy. Increased serum or lesional levels of specific interleukins do not ensure the efficacy of the corresponding biologic. As seen in alopecia areata and vitiligo, numerous studies documented increased TNF-α and IL-17 levels while the inhibition of both cytokines failed in these disorders (10, 11). Unfortunately, the lack of convincing mouse models for most dermatologic disorders limits the evidence that can be obtained before conducting a clinical trial.

Getting biologics approved and reimbursed for these new indications is another challenge. Weighing economic implications vs. health improvement will remain a difficult balance in the next decades. The rise of biosimilars may improve access to off-label use in disorders which are unlikely to be of economic interest due to their rare incidence or limited patients with severe disease requiring biologics (109). An increased appreciation and funding of investigator-initiated clinical trials seem of particular relevance to explore the full capacity of biologics.

## Author Contributions

RS reviewed the literature, drafted the manuscript, and made the figures. JL and NvG critically revised the manuscript.

### Conflict of Interest Statement

The authors declare that the research was conducted in the absence of any commercial or financial relationships that could be construed as a potential conflict of interest.
