# Supplementary material for: Learning From Success and Failure: Biologics for Non-approved Skin Diseases
Source: Front Immunol. 2019 Aug 8;10:1918. doi: 10.3389/fimmu.2019.01918 (PMC6694799; doi:10.3389/fimmu.2019.01918)
Supplement: Supplementary file 1 [file Table_1.DOCX]

**Supplementary Table S1: Pathway, type, effect and characteristics of biologics used off-label for skin disorders**

| **IL-1** | | | | |
| --- | --- | --- | --- | --- |
| **Role in health** | | **Role in skin disease** | | **Immunological effects** |
| - Major mediator of innate immune response | | - Crucial trigger of autoinflammatory diseases | | - Induction of IL-1 target genes (IL-6, IL-8, MCP-1, COX-2…) (1) |
| Biologic | Type | | Differences between biologics | |
| Anakinra | Recombinant IL-1 receptor antagonist | | - Anakinra competes with IL-1α and IL-1β to bind to the IL-1 receptor.  - Canakinumab inhibits IL-1β and has a longer half-life (26d) compared to anakinra (4-6h) (2). | |
| Canakinumab | Monoclonal antibody (MoAb) against IL-1β | |  |  |
| **IL-17** | | | | |
| **Role in health** | | **Role in skin disease** | | **Immunological effects** |
| - Epithelial barrier defence against fungal infections, candida and bacteria | | - Key factor in psoriasis and psoriasis arthritis  - Plausible role in neutrophilic disorders | | - Acts on non-hematopoietic cells to promote keratinocyte proliferation  - initiates a feed-forward amplification loop promoting additional IL-17 signaling (3)  - Synergistic effect on other cytokines (e.g. TNF-α) (4) |
| Biologic | Type | | Differences between biologics | |
| Secukinumab | MoAb against IL-17A | | - Secukinumab and ixekizumab inhibit both IL-17A.  - Brodalumab inhibits IL-17A, IL-17F, IL-17C and IL-17E | |
| Ixekizumab |  |  |  |  |
| Brodalumab | MoAb against the IL-17 receptor A subunit | |  |  |
| **IL-12** | | | | |
| **Role in health** | | **Role in skin disease** | | **Immunological effects** |
| - Stimulating Th1-mediated immunity (antiviral and antitumoral protection) | | - Increased levels are found in psoriasis skin (5), although the contributing role of IL-12 is unclear in psoriasis (6) | | - Stimulates differentiation of naive T cells into Th1 cells  - Inducer of IFN-γ in NK cells and T cells (7) |
| Biologic | Type | | Differences between biologics | |
| Ustekinumab | MoAb against the p40 subunit common to IL-12 and IL-23 | | - Ustekinumab interacts with both the Th1 and Th17 pathway by blocking IL-12 and IL-23 | |
| **IL-23** | | | | |
| **Role in health** | | **Role in skin disease** | | **Immunological effects** |
| - Regulation of the IL-17 pathway | | - ‘Upstream’ cytokine elevated in psoriasis and other Th17 mediated disorders | | - Acts on hematopoietic cells.  - Late factor in the differentiation of Th17 cells |
| Biologic | Type | | Differences between biologics | |
| Guselkumab | MoAb against the p19 subunit of IL-23 | | - Anti-p19 antibodies do not inhibit IL-12 which might lead superior efficacy with less adverse events in IL-23/IL-17 mediated disorders such as psoriasis (8) | |
| Risankizumab |  |  |  |  |
| Tildrakizumab |  |  |  |  |
| **TNF-α** | | | | |
| **Role in health** | | **Role in skin disease** | | **Immunological effects** |
| - Widely present pro-inflammatory cytokine  - Protective against infections (9) | | - Role in psoriasis, hidradenitis suppurativa and granuloma annulare is evident. | | - Stimulates IL-23 production by dendritic cells  - Synergises with other cytokines (e.g. IL-17, IFN-γ) (10,11) |
| Biologic | Type | | Differences between biologics | |
| Adalimumab | MoAb against TNF-α | | - Most data are available for adalimumab and etanercept.  - The lack of an Fc-region in certolizumab pegol minimizes complement and antibody dependent cell-mediated cytotoxicity and active transfer over the placenta (12)  - Infliximab is administered intravenously (>< subcutaneously) and is less frequently used in dermatology. | |
| Infliximab |  |  |  |  |
| Certolizumab Pegol | MoAb, Fc-free, PEGylated, against TNF-α | |  |  |
| Etanercept | TNF receptor linked to the Fc portion of human IgG1 | |  |  |
| **IgE** | | | | |
| **Role in health** | | **Role in skin disease** | | **Immunological effects** |
| - Eradication of parasitic infections | | - Allergy and urticaria  - Bullous pemphigoid | | - Mast cell degranulation  - Eosinophil chemotaxis |
| Biologic | Type | | Differences between biologics | |
| Omalizumab | MoAb against IgE | | - Omalizumab binds stronger to free IgE than the high affinity IgE receptor (13) | |

**References**

1. Weber A, Wasiliew P, Kracht M. Interleukin-1 (IL-1) pathway. *Sci Signal* (2010) **3**:cm1. doi:10.1126/scisignal.3105cm1

2. Kontzias A, Efthimiou P. The Use of Canakinumab, a Novel IL-1β Long-Acting Inhibitor, in Refractory Adult-Onset Still’s Disease. *Seminars in Arthritis and Rheumatism* (2012) **42**:201–205. doi:10.1016/j.semarthrit.2012.03.004

3. Amatya N, Childs EE, Cruz JA, Aggor FEY, Garg AV, Berman AJ, Gudjonsson JE, Atasoy U, Gaffen SL. IL-17 integrates multiple self-reinforcing, feed-forward mechanisms through the RNA binding protein Arid5a. *Sci Signal* (2018) **11**: doi:10.1126/scisignal.aat4617

4. Veldhoen M. Interleukin 17 is a chief orchestrator of immunity. *Nat Immunol* (2017) **18**:612–621. doi:10.1038/ni.3742

5. Yawalkar N, Karlen S, Hunger R, Brand CU, Braathen LR. Expression of interleukin-12 is increased in psoriatic skin. *J Invest Dermatol* (1998) **111**:1053–1057. doi:10.1046/j.1523-1747.1998.00446.x

6. Kulig P, Musiol S, Freiberger SN, Schreiner B, Gyülveszi G, Russo G, Pantelyushin S, Kishihara K, Alessandrini F, Kündig T, et al. IL-12 protects from psoriasiform skin inflammation. *Nat Commun* (2016) **7**:13466. doi:10.1038/ncomms13466

7. Vignali DAA, Kuchroo VK. IL-12 family cytokines: immunological playmakers. *Nat Immunol* (2012) **13**:722–728. doi:10.1038/ni.2366

8. Tonini A, Gualtieri B, Panduri S, Romanelli M, Chiricozzi A. A new class of biologic agents facing the therapeutic paradigm in psoriasis: anti-IL-23 agents. *Expert Opin Biol Ther* (2018) **18**:135–148. doi:10.1080/14712598.2018.1398729

9. Murdaca G, Spanò F, Contatore M, Guastalla A, Penza E, Magnani O, Puppo F. Infection risk associated with anti-TNF-α agents: a review. *Expert Opin Drug Saf* (2015) **14**:571–582. doi:10.1517/14740338.2015.1009036

10. Goldberg M, Nadiv O, Luknar-Gabor N, Agar G, Beer Y, Katz Y. Synergism between tumor necrosis factor alpha and interleukin-17 to induce IL-23 p19 expression in fibroblast-like synoviocytes. *Molecular Immunology* (2009) **46**:1854–1859. doi:10.1016/j.molimm.2009.01.004

11. Mehta NN, Teague HL, Swindell WR, Baumer Y, Ward NL, Xing X, Baugous B, Johnston A, Joshi AA, Silverman J, et al. IFN-γ and TNF-α synergism may provide a link between psoriasis and inflammatory atherogenesis. *Scientific Reports* (2017) **7**:13831. doi:10.1038/s41598-017-14365-1

12. Goel N, Stephens S. Certolizumab pegol. *MAbs* (2010) **2**:137–147.

13. Belliveau PP. Omalizumab: A Monoclonal Anti-IgE Antibody. *MedGenMed* (2005) **7**:27.
